# Oral Chinese Herbal Medicine as Prophylactic Treatment for Episodic Migraine in Adults: A Systematic Review and Meta-Analysis of Randomized Controlled Trials

**DOI:** 10.1155/2020/5181587

**Published:** 2020-12-28

**Authors:** Shaohua Lyu, Claire Shuiqing Zhang, Xinfeng Guo, Anthony Lin Zhang, Jingbo Sun, Chuanjian Lu, Charlie Changli Xue, Xiaodong Luo

**Affiliations:** ^1^The Second Affiliated Hospital of Guangzhou University of Chinese Medicine, Guangdong Provincial Hospital of Chinese Medicine and Guangdong Provincial Academy of Chinese Medical Sciences, Guangzhou 510120, China; ^2^The China-Australia International Research Centre for Chinese Medicine, School of Health and Biomedical Sciences, Royal Melbourne Institute of Technology, Melbourne 3083, Australia

## Abstract

**Background:**

The prophylactic effects of Chinese herbal medicine (CHM) for migraine were examined in numerous clinical trials. This review aimed to analyze the effectiveness and safety of CHM as prophylactic treatment of migraine compared to flunarizine.

**Methods:**

Nine databases were searched for randomized controlled trials (RCTs) that evaluated effects of CHM for episodic migraine prophylaxis compared to flunarizine, published before March 2019.

**Results:**

Thirty-five RCTs with 2,840 participants met the inclusion criteria, and 31 of them were included in meta-analyses. The overall meta-analysis indicated that, when compared to flunarizine, CHM reduced the frequency of migraine attacks at the end of treatment (EoT) (21 studies, mean difference (MD) −1.23, 95% confidence interval (CI) (−1.69, −0.76)) and at the end of follow-up (EoFU) (five studies, MD −0.96, 95% CI (−1.70, −0.21)). Subgroup analyses based on the treatment duration, follow-up duration, and the dosage of flunarizine showed that CHM was superior to or comparable with flunarizine in reducing migraine frequency. Similar results were also found for secondary outcomes such as the pain visual analogue scale, migraine duration, responder rate, and acute medication usage. In particular, the studies that used CHM containing herb pairs (Chuan Xiong plus Bai Zhi and Chuan Xiong plus Tian Ma) showed promising results. However, the certainty of this evidence was evaluated as “low” or “very low” using the Grading of Recommendations, Assessment, Development and Evaluations approach.

**Conclusion:**

CHM appeared to be comparable with flunarizine in reducing the frequency of episodic migraine attacks in adults at EoT and EoFU and well-tolerated by participants, regardless of the treatment duration, follow-up duration, and dosage of flunarizine. Due to the low certainty of the evidence, the suggested promising prophylactic outcomes require higher quality evidence from further rigorous RCTs.

## 1. Introduction

Migraine is a primary headache disorder, manifesting in episodic headache attacks which usually lasts for 4–72 hours. The typical characteristics of a migraine headache include unilateral, pulsating pain with moderate to severe intensity, aggravation by routine physical activity, and association with nausea and/or photophobia and phonophobia [1].

Migraine has an estimated global prevalence of 14.7% [2]. According to the Global Burden of Disease published in 2018, migraine was ranked as the seventh most disabling disease and the third leading cause of disability of people aged 15–49 years [3]. Furthermore, the total financial burden of the disease on individuals and society, as reported in 2010, equated to over three billion pounds a year in the United Kingdom [4].

Migraine can be subdivided into episodic and chronic migraine. The former refers to headache attacks occurring less than 15 days per month, while the latter refers to 15 or more headache days per month. Episodic migraine accounts for the majority of migraineurs [1], but the condition can progress to chronic migraine, if not properly managed [5]. It was estimated that approximately 2.5% of episodic migraine cases develop into chronic migraine annually [6].

The clinical management of migraine involves pain rescue and prophylactic treatment. Generally, migraineurs are advised to be on continuous prophylactic treatment to reduce the frequency and severity of attacks. However, it was estimated that more than half of the migraineurs were unsatisfied with prophylactic pharmacotherapy due to insufficient improvements and unbearable side effects [7–9]. Flunarizine is a first-line medication recommended by clinical guidelines for migraine prophylaxis [10–13]. Its effects of preventing migraine attacks in adults have been confirmed by recently published systematic reviews [14, 15], clinical trials [16–18], and experimental studies [19–23]. However, unwanted adverse effects such as tiredness, mood swings, weight gain, and depression limit its use in clinical practice [16, 24].

Chinese herbal medicine (CHM) has been widely used in clinical practice for thousands of years in China, usually in the form of herbal formulas consisting of a group of individual herbs and often involves herb pairs [25]. CHM has been gradually gaining acceptance worldwide [26–29]. There have been a number of laboratory experiments [30–32], clinical trials [33], and systematic reviews with meta-analyses [34–37] evidence supporting CHM as a potential alternative therapy for migraine.

Four systematic reviews showed that oral CHM was more effective than placebo or conventional pharmacotherapies for migraine management [35–38]. However, none of these reviews provided evidence of prolonged treatment effects, which is important for migraine prophylaxis. In addition, the reviews accepted a range of medication used in the control groups [35–37]. Furthermore, most of the studies included in these reviews did not fulfil requirement that an effective migraine prophylaxis should be taken for no less than four weeks [10–12, 39, 40].

To provide more precise evidence to support the use of oral CHM for preventing episodic migraine in adults, this systematic review evaluates the clinical effectiveness and safety of CHM comparing with a first-line medication (flunarizine) in randomized controlled trials (RCTs).

## 2. Methods

This review applies the methods recommended by the Cochrane Handbook of Systematic Reviews of Interventions 5.1.0 [41]. The review protocol was registered in PROSPERO (CRD42019123039).

### 2.1. Database Search and Study Screening

A rigorous electronic search was initially conducted in five English databases—PubMed, Excerpta Medica Database (EMBASE), Cumulative Index of Nursing and Allied Health Literature (CINAHL), Cochrane Central Register of Controlled Trials (including the Cochrane Library), and the Allied and Complementary Medicine Database (AMED), and four Chinese databases—Biomedical Literature, China National Knowledge Infrastructure (CNKI), Chongqing VIP (CQVIP), and Wanfang database, from their inceptions to November 2018. An updated search was conducted in March 2019. The search strategy was designed according to three groups of search terms: participant condition (migraine), intervention (Chinese medicine, CHM, and related terms), and control (flunarizine). Reference lists of previously published reviews were screened for eligibility.

### 2.2. Study Selection

The inclusion criteria for this review were as follows: (1) participants aged between 18 and 75 years; (2) diagnosis of episodic migraine, with or without aura, according to clinical guidelines [1, 39, 40, 42]; (3) treatment intervention of orally administered CHM; (4) utilized only flunarizine as the control intervention; studies which allowed acute pain medications were included if the same medications were used in both the intervention and control groups; and (5) evaluated at least one of the following outcomes: migraine frequency, number of migraine days per month, responder rate, headache pain severity, average duration of attack, acute medication usage, and health-related quality of life.

The exclusion criteria were as follows: (1) studies which focused on acute migraine attack management; (2) combination of CHM with other types of Chinese medicine therapy or pharmacotherapy; (3) treatment duration of less than four weeks; and (4) different acute pain rescue medications applied in the intervention and control groups.

### 2.3. Data Extraction

After screening titles and abstracts, full texts were obtained and checked for eligibility by two authors (SL and CSZ). Data from eligible studies were extracted and cross-checked by two research assistants (YX and LL) using the EpiData software (EpiData Association, Odense, Denmark). Information of authors, publication year, title, journal, setting, study design, diagnostic criteria, sample size, dropout, age, gender, duration of migraine, CHM formula names and ingredients, dosage of flunarizine, treatment duration, follow-up duration, outcome measures, and adverse events (AEs) were extracted. Disagreements were discussed and resolved by the reviewers (SL and CSZ). Where there were missing, conflicting, or unclear data, we contacted the authors of the respective studies for clarification.

### 2.4. Risk of Bias Assessment

The methodological quality of included studies was assessed by two authors (SL and CSZ) using the Cochrane Risk of Bias Tool [41]. Trials were judged as “low,” “unclear,” or “high” risk of bias for the domains of sequence generation, allocation concealment, blinding of participants, blinding of personnel and outcome assessors, incomplete outcome data, selective reporting, and other forms of bias such as conflicts of interest. Discrepancies were discussed with a third reviewer (XG).

### 2.5. Publication Bias Assessment

Publication bias was assessed by funnel plots and Egger's test using the Stata 12 software (StataCorp LLC, Texas, USA), where more than 10 RCTs were included in the meta-analysis.

### 2.6. Certainty of Evidence Assessment

The certainty of evidence, referring to the strength or reliability of study findings, was evaluated using the Grading of Recommendations, Assessment, Development and Evaluations (GRADE) approach [43, 44]. The GRADE approach classifies the certainty of evidence in four levels (high, moderate, low, and very low) based on five factors: risk of bias, imprecision, inconsistency, indirectness, and publication bias.

### 2.7. Data Analyses

Available data were merged for meta-analyses in RevMan 5.3.0 to evaluate the effects of CHM. The primary outcome measure was the frequency of migraine, and the secondary outcomes included days of migraine, pain visual analogue scale (VAS), duration of migraine attack, responder rate, acute medication usage, quality of life scores, and AEs. Treatment effects were evaluated at two time points: at the end of treatment (EoT) and at the end of follow-up (EoFU), where possible. Frequency analyses were conducted on CHM formulas and individual herbs. Mean difference (MD) and 95% confidence intervals (CI) were used for continuous data, while risk ratios (RR) with 95% CIs were for dichotomous data. Statistical heterogeneity was assessed using the *I*^2^ statistic. The random-effects model was selected for the meta-analyses presenting high heterogeneity with unknown reason; otherwise, the fixed-effects model was employed [41]. Where possible, subgroup analyses were performed to explore heterogeneity based on variables including the treatment duration, follow-up duration, and the dosage of flunarizine. Subgroup analyses were also conducted based on RCTs which applied the same CHM formulas and common herb pairs. AEs were summarized, and the frequencies were compared between groups.

## 3. Results

**Figure 1:**
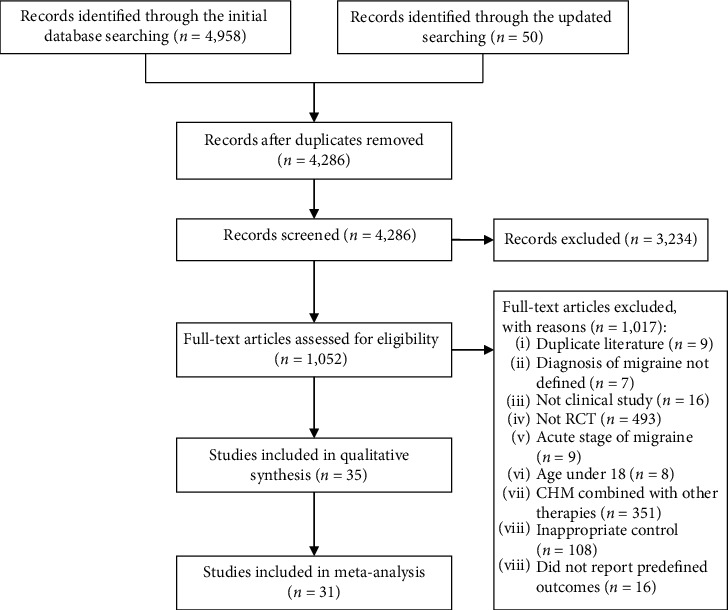
PRISMA flowchart of the study selection process.

The original comprehensive electronic database search (until November 2018) identified 4,958 citations, and the updated search conducted in March 2019 yielded another 50 citations. In total, 35 RCTs met the inclusion criteria, with 31 RCTs included in the meta-analyses (Figure 1).

### 3.1. Characteristics of Included Studies

All included studies were open-label studies conducted in China and published in the Chinese language from 2003 to 2019. The RCTs enrolled 2,840 participants, with sample size ranging from 32 [45] to 240 [46] people. Dropouts were reported in seven studies [46–52]. The age of participants ranged from 18 to 75 years old, with disease durations between one month and 38 years. All studies used either 5 mg or 10 mg flunarizine in control groups and allowed acute pain rescue medicine as needed. According to the available information on gender, there were more female than male (1,750 vs. 1,060), but none of the studies reported gender-based treatment effects data. The treatment duration ranged from 28 days to 90 days. Eight studies involved a follow-up phase [46, 50, 53–58], where six provided detailed outcome data [46, 50, 53–56] (Table 1).

The outcome measures reported by the included studies were migraine frequency, migraine attack duration, migraine days, pain VAS, responder rate, usages of pain medication, and quality of life using the 6-item Headache Impact Test (HIT-6). With regard to AEs, nine studies [49, 52, 54, 56, 59–63] provided a general statement that there were no severe AEs, 15 studies reported details of AEs [47, 48, 50, 55, 57, 58, 71–79], and the remaining eleven studies did not report information on AEs [45, 46, 51, 53, 64–70].

### 3.2. CHM Treatments Used in the Included Studies

CHM was administered in the forms of decoctions (28 studies), capsules (five studies) [47, 53, 55, 57, 72], granules (one study) [78], and pills (one study) [71] (Table 1). Twenty-seven CHM formulas involving 104 individual herbs were used in the included studies. Two formulas were evaluated by multiple studies, namely, San Pian Tang [50, 74] and Zheng Tian granules [55] or pills [71]. The most frequent herbs used by all studies were Chuan Xiong (31 studies), Bai Zhi (18 studies), Bai Shao (16 studies), Gan Cao (13 studies), Tian Ma (13 studies), Dang Gui (12 studies), and Chai Hu (11 studies) (Table 2). It should be noted that, two herb pairs were frequently used by the included studies, specifically Chuan Xiong plus Bai Zhi (18 studies) and Chuan Xiong plus Tian Ma (10 studies). These two herb pairs had been developed into commercialized CHM products for migraine and documented in the Pharmacopoeia of China [80].

### 3.3. Risk of Bias

**Figure 2:**
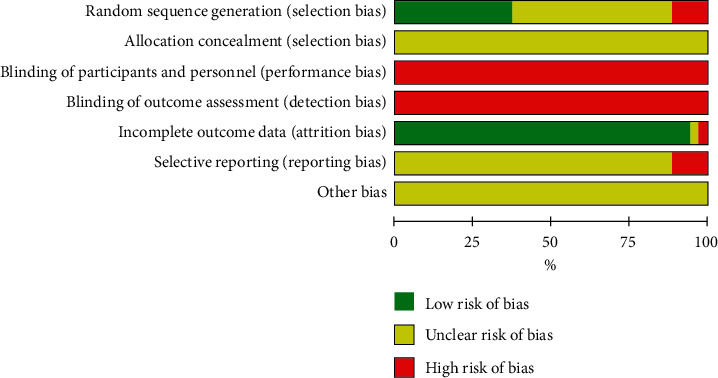
Risk of bias assessment of the included RCTs. RCT, randomized control trial.

All studies mentioned randomization, however, only 13 RCTs (37.1%)[46, 48, 49, 51, 55, 59, 60, 62, 67, 69, 76, 78, 79] were assessed to have “low risk” of bias in terms of sequence generation; four studies (11.4%) [45, 50, 54, 74] were considered as “high risk” in this domain as participants were allocated based on the order of enrollment. All included studies were assessed as “unclear risk” of bias for allocation concealment due to lack of adequate information. In terms of blinding of participants, research personnel, and outcome assessors, all studies were judged as “high risk” of bias because no adequate blinding methods were employed despite the different types of intervention between groups. Most of the studies were at “low risk” of bias for incomplete outcome data; only one [48] was assessed as “high risk” in this domain due to a high and unbalanced dropout rate. Thirty-one studies (88.6%) were assessed as “unclear risk” of bias regarding selective outcome reporting due to the lack of registered protocols, while four RCTs were assessed as “high risk” because they did not report AEs [53] or outcome data of the follow-up phase [56–58] (Figure 2).

### 3.4. Treatment Effects

#### 3.4.1. Primary Outcomes Measures

*(1) Frequency of Migraine at EoT*. Twenty-one studies with 1,567 participants reported the frequency of migraine attacks at EoT. Overall meta-analysis showed that oral CHM was more effective than flunarizine in terms of reducing migraine attack frequency (MD: −1.23, 95% CI (−1.69, −0.76), *I*^2^ = 97%).

**Figure 3:**
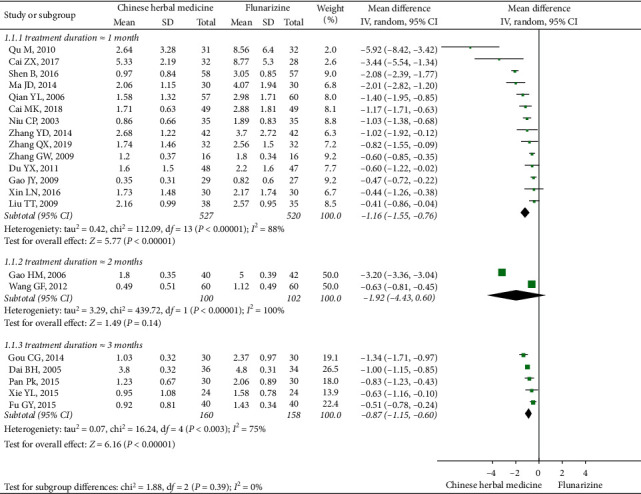
Subgroup meta-analysis results of migraine frequency at the end of treatment based on treatment duration.

The subgroup analysis based on treatment duration indicated that CHM was superior to flunarizine when applied for a treatment period of 28 or 30 days (14 studies, MD: −1.16, 95% CI (−1.55, −0.76), *I*^2^ = 88%) [45, 47, 48, 50, 56, 58–61, 66, 67, 69, 70, 78] and 84 or 90 days (five studies, MD: −0.87, 95% CI (−1.15, −0.60), *I*^2^ = 75%) [53–55, 64, 74]. However, such effects were not seen in the subgroup of RCTs conducting 56 or 60 days treatments (two studies, MD: −1.92, 95% CI (−4.43, 0.60), *I*^2^ = 100%) [57, 77] (Figure 3).

**Figure 4:**
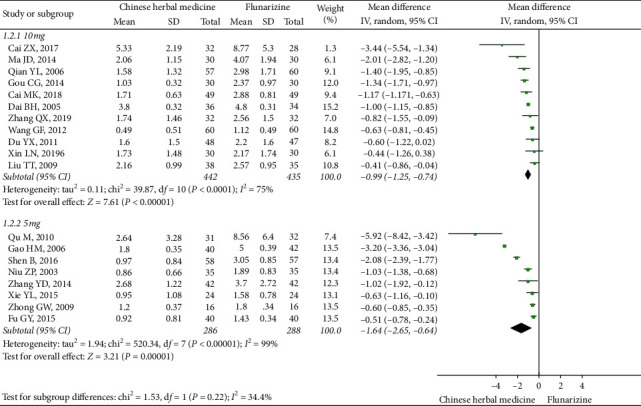
Subgroup meta-analysis results of migraine frequency at the end of treatment based on flunarizine dosage.

The subgroup analysis based on dosage of flunarizine showed that oral CHM was more effective than flunarizine on both 5 mg daily (eight studies, MD: -1.64, 95% CI (−2.65, −0.64), *I*^2^ = 99%) [45, 50, 55, 57, 64, 66, 67, 70] and 10 mg daily (11 studies, MD: −0.99, 95% CI (−1.25, −0.74), *I*^2^ = 75%) [47, 48, 53, 56, 59–61, 69, 74, 77, 78] (Figure 4).

**Figure 5:**
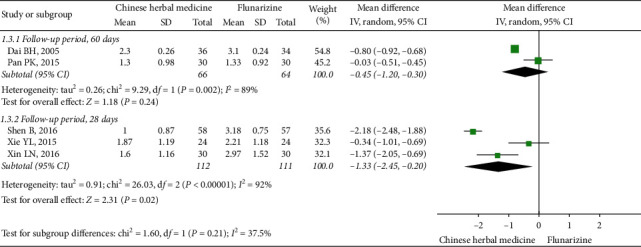
Subgroup meta-analysis results of migraine frequency at the end of follow-up based on follow-up duration.

*(2) Frequency of Migraine at EoFU*. Five studies reported the frequency of migraine attacks at EoFU. The follow-up duration was either 28 days [50, 55, 56] or 60 days [53, 54]. The overall meta-analysis favored CHM (MD: −0.96, 95% CI (−1.70, −0.21), *I*^2^ = 96%). Subgroup analyses showed CHM being more effective than flunarizine at the end of a 28-day follow-up phase (three studies, MD: −1.33, 95% CI (−2.45, −0.20), *I*^2^ = 92%) [50, 55, 56], but not at the end of a 60-day follow-up phase (two studies, MD: −0.45, 95% CI (−1.20, 0.30), *I*^2^ = 89%) [53, 54] (Figure 5).

**Figure 6:**
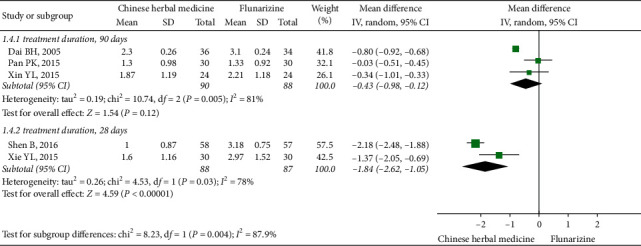
Subgroup meta-analysis results of migraine frequency at the end of follow-up based on treatment duration.

Two RCTs which conducted a 28-day treatment showed that at EoFU, CHM was more effective than flunarizine (MD: −1.84, 95% CI (−2.62, −1.05), *I*^2^ = 78%) [50, 56]. While studies which conducted a 90-day treatment showed no difference between groups at EoFU (three studies, MD: −0.43, 95% CI (−0.98, 0.12), *I*^2^ = 81%) [53–55] (Figure 6).

**Figure 7:**
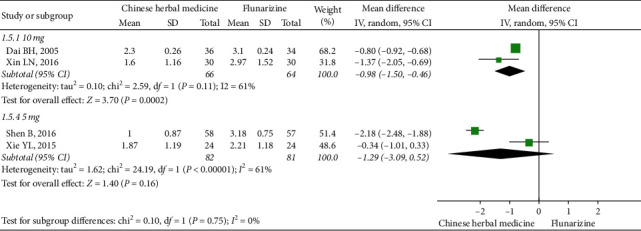
Subgroup meta-analysis results of migraine frequency at the end of follow-up based on flunarizine dosage.

Based on the dosage of flunarizine, it was found that CHM showed equivalent effects when compared to a dose of 5 mg flunarizine at EoFU (two studies, MD: −1.29, 95% CI (−3.09, 0.52), *I*^2^ = 96%) [50, 55], but was more effective when compared with 10 mg daily flunarizine (two studies, MD: −0.98, 95% CI (−1.50, −0.46), *I*^2^ = 61%) [53, 56] (Figure 7).

#### 3.4.2. Secondary Outcomes Measures

*(1) Days of Migraine*. Four studies with 446 participants [46, 50, 55, 56] reported data on the days of migraine attack, and the meta-analysis showed no difference between CHM and flunarizine, both at EoT (MD: −1.65 (−3.85, 0.54), *I*^2^ = 96%) and EoFU (MD: −2.18 (−5.08, 0.72), *I*^2^ = 97%) (Table 3).

*(2) Pain VAS*. Fourteen studies with 1,036 participants [48–50, 52, 55, 64, 68, 71–76, 78] reported pain VAS at EoT, showing a greater pain reduction achieved by CHM than flunarizine (MD: −1.04 (−1.67, −0.40), *I*^2^ = 96%). However, there was no difference at EoFU of 28 days (two studies, MD: −1.56 (−3.73, 0.61), *I*^2^ = 96%) [50, 55] (Table 3).

*(3) Duration of Migraine Attack*. Twenty studies with 1,495 participants reported the average duration of migraine attacks [45, 47, 48, 51, 53–55, 57, 58, 60, 64, 66, 69, 70, 72, 74, 75, 77–79]. Oral CHM was more effective than flunarizine in shortening the duration of migraine attacks at EoT (MD: −2.24 (−3.18, −1.30), *I*^2^ = 92%) but not at EoFU (three studies, MD: −3.60 (−8.85, 1.66), *I*^2^ = 97%) [53–55] (Table 3).

*(4) Responder Rate*. Five studies [46, 55, 56, 65, 75] involving 547 participants reported responder rate at EoT and meta-analysis showed superior effects of CHM (RR: 1.37 (1.23, 1.52), *I*^2^ = 0%) (Table 3).

*(5) Acute Medication Usage*. In terms of the acute medication usage, participants in the CHM group used less pain medication than those in the flunarizine group at both EoT (five studies, MD: −0.58 (−1.03, −0.13), *I*^2^ = 94%) [46, 50, 54, 55, 59] and EoFU (four studies, MD: −0.69 (−1.22, −0.15), *I*^2^ = 96%) [46, 50, 54, 55] (Table 3).

*(6) Quality of Life*. One study [62] involving 120 participants reported data on quality of life using HIT-6 at EoT, with the results favoring the CHM group (Table 3).

#### 3.4.3. Meta-Analyses for Individual Formula

Two RCTs [50, 74] evaluated the effectiveness of the oral CHM formula San Pian Tang on 175 participants. Meta-analysis of pain VAS showed that San Pian Tang was more effective than flunarizine at EoT (MD: −1.88, 95% CI (−3.14, −0.62), *I*^2^ = 92%). Another two studies with 108 participants [55, 71] evaluated the effectiveness of Zheng Tian pills/granules in reducing pain VAS at EoT, showing that Zheng Tian pill/granule achieved lower pain VAS than flunarizine at EoT (MD: −0.64, 95% CI (−1.08, −0.20), *I*^2^ = 0%) (Table 3).

#### 3.4.4. Frequency of Migraine Based on Herb Pairs

Common herb pairs identified from the CHM formulas of the RCTs were pooled for subgroup analyses for migraine frequency. Ten RCTs [48, 50, 54–56, 60, 61, 64, 74, 77] with 793 participants used CHM containing the herb pair Chuan Xiong plus Bai Zhi. These studies achieved superior effects of CHM in reducing migraine attack frequency at EoT (MD: −1.00, 95% CI (−1.41, −0.60), *I*^2^ = 90%). However, such effects were not observed at EoFU (four studies, MD: −0.99, 95% CI (−2.17, 0.19), *I*^2^ = 96%) [50, 54–56] (Table 3).

Four studies used the herb pair Chuan Xiong plus Tian Ma [45, 57, 64, 70] and showed no difference between two groups at EoT (MD: −1.34, 95% CI (−3.00, 0.32), *I*^2^ = 99%) (Table 3).

### 3.5. Publications Bias

**Figure 8:**
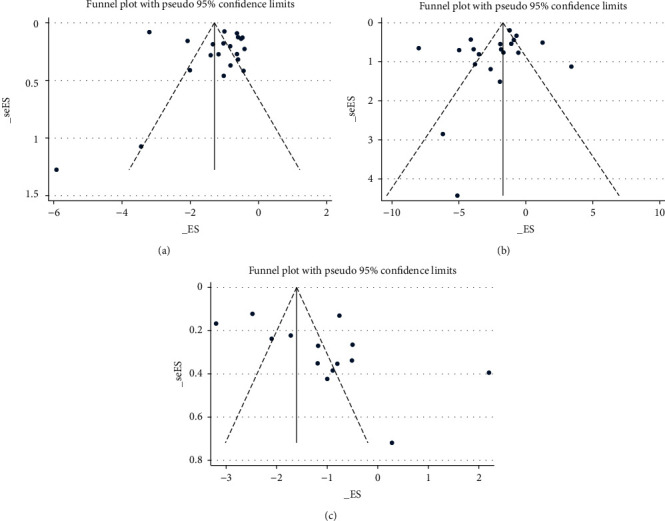
Funnel plot. (a) Migraine frequency at the end of treatment. (b) Migraine attack duration at the end of treatment. (c) Pain VAS at the end of treatment; VAS, visual analogue scale.

**Figure 9:**
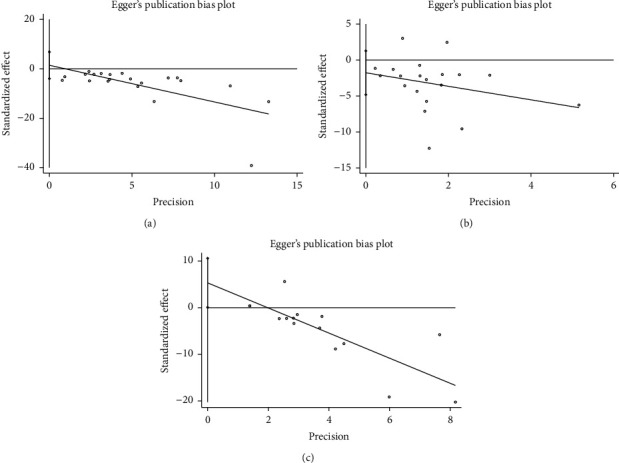
Egger's test. (a) Migraine frequency at the end of treatment. (b) Migraine attack duration at the end of treatment. (c) Pain VAS at the end of treatment; VAS, visual analogue scale.

The funnel plots of migraine frequency, migraine attack duration, and pain VAS at EoT were conducted as the meta-analyses of these outcomes involved more than ten studies. All funnel plots (Figure 8) were symmetrical and seemed unlikely to have publication bias. Egger's test was further conducted, and publication bias was not detected (*P* > 0.05) (Figure 9). Funnel plots and Egger's test could not be conducted for the other outcome measures due to the limited number of included studies.

### 3.6. Assessment Using GRADE

The certainty of evidence obtained from meta-analyses on the primary outcome measures is presented in Table 4. Oral CHM was more effective than flunarizine for reducing migraine frequency at EoT and EoFU, but the certainty of this evidence was “low” and “very low,” respectively.

### 3.7. Adverse Events

AEs were categorized and calculated to assess the safety of the treatments. Based on the available data from 15 studies that reported detailed information of AEs, the number of AEs in the CHM group was less than that in the flunarizine group (34 vs 50). As shown in Table 5, most AEs were mild and did not require additional medical management. None of the participants dropped out due to AEs. Gastrointestinal symptoms (i.e., nausea, stomach discomfort, diarrheal, and abdominal distension) and other symptoms (including drowsiness, dizziness, fatigue, and insomnia) were commonly seen in both CHM and flunarizine groups. Other AEs reported in the CHM group were three cases of irregular menstruation [57], one acne [48], and one slight decrease in platelet count [55], while the flunarizine group had common reports of weight gain [57, 76], some extrapyramidal symptoms such as ataxic [57, 72] and involuntary movements [47], as well as one case of moderate liver function impairment [76].

## 4. Discussion

### 4.1. Summary of Results

This systematic review provides evidence showing that oral CHM is more effective than flunarizine for episodic migraine prophylaxis based on these outcome measures: migraine frequency, pain VAS, migraine attack duration, responder rate, and acute medication usage at EoT. Oral CHM also showed better effects than flunarizine for migraine frequency and acute medication usage at EoFU. In addition, there was no difference between CHM and flunarizine in migraine days (at both EoT and EoFU) and pain VAS and migraine attack duration at EoFU. Nevertheless, the overall methodological quality of the included studies was low, and the certainty of evidence was “low” or “very low” based on GRADE assessment.

As substantial heterogeneity existed in most meta-analyses, subgroup meta-analyses were conducted on the primary outcome measures, based on the treatment duration, follow-up duration, and dosage of flunarizine. These subgroup analyses showed that CHM produced superior or equivalent effects as flunarizine. However, heterogeneity remained considerable within the subgroup analyses. The possible causes of heterogeneity are the uses of different CHM formulas, disease severity, and duration across trials. It should be noted that two studies reported migraine frequency at EoT with exceptional results [51, 75]; therefore, they were excluded from the meta-analysis of this outcome.

This study shows that CHM is well-tolerated when compared to flunarizine. Fifteen studies provided detailed information on AEs; all of them were mild or moderate and did not require specific management. Most of the common complaints in both CHM and flunarizine groups such as fatigue, insomnia, and digestive problems could be the associated symptoms of migraine [1] rather than side effects caused by treatments. One case of decreased platelet count was reported, but there was not a confirmed association between this event and CHM [55]. Weight gain and ataxia were only reported in the flunarizine group, which agrees with previous research results on flunarizine [15, 16, 18, 24, 81, 82]. Considering that aging [81, 83–85] and increased dosage [11, 40, 86, 87] of flunarizine and the predisposing factors for its side effects, CHM could be an alternative for elderly patients and those who suffer from common side effects of flunarizine.

As shown in the meta-analyses, CHM has an advantage in reducing acute pain medication usage, indicating its potential of preventing medication overuse, which is a common concern in headache [10] and chronic migraine [88] treatment.

Previous research suggested that CHM is an effective add-on therapy for migraine [36]. It is known that flunarizine should be taken for several weeks to show its full effects in migraine prevention [11, 40, 83, 84]; during this period, patients may be unsatisfied with its treatment effects. The subgroup analysis in our review demonstrated that oral CHM was superior to flunarizine when they were used for four weeks and equivalent to flunarizine when they were used for eight weeks. Hence, our results support the use of CHM as a potential adjuvant therapy to increase the effectiveness of flunarizine. However, the drug interactions between flunarizine and Chinese medicine herbs or formulas have not been well investigated, and this is an area which requires further research.

### 4.2. Mechanisms of Herbs

There has been increasing experimental research on the active compounds of CHM in attempts to elucidate their potential mechanisms for migraine. For example, one of the main compounds found in Chuan Xiong (*Ligusticum chuanxiong*), senkyunolide I, was proved to reduce migraine pain by adjusting the levels and turnover rates of monoamine neurotransmitters and decrease nitric oxide (NO) levels in the blood and brain [32]. Bai Zhi (Angelicae Dahuricae Radix) was reported to have antimigraine actions by modulating the levels of vasoactive substances such as NO, calcitonin gene-related peptides, and endothelin [89, 90]. Tian Ma (*Gastrodia elata*) contains the active ingredient, gastrodin, which has been found to demonstrate antimigraine, antihyperalgesic, and antinociceptive effects, possibly by inhibiting trigeminal nerve activation at central sites and also inhibiting the peripheral release of calcitonin gene-related peptides following the NO scavenging effect [91, 92].

This review has also provided meta-analysis evidence supporting the use of two herb pairs in migraine, namely, Chuan Xiong plus Bai Zhi and Chuan Xiong plus Tian Ma. Herb pairs form the basis of CHM formulation and are believed to result in synergistic effects or reduced side effects/toxicity [93]. It has been suggested that herb pairs are potential research entry-point for research on CHM mechanisms [93]. The two herb pairs evidenced in this review are also CHM formulas that have been traditionally used for the treatment of headaches/migraines [80]. The combination of Chuan Xiong and Bai Zhi is a formula known as Du Liang Wan with experimental studies showing the function of adjusting the level of neurotransmitters and vasoactive substances to relieve neurogenic inflammation [94, 95]. The other herb pair (Chuan Xiong plus Tian Ma) is known as Da Chuan Xiong Wan, which has been proved to reduce inflammatory mediators through inhibition of the NF-kappaB pathway [31].

### 4.3. Strengths and Limitations of This Study

One strength of this review is that the prolonged effects of oral CHM has been evaluated, which has been highlighted by clinical guidelines as an important outcome assessment of prophylactic treatments of migraine [10, 11, 96, 97]. Furthermore, the active comparator in this review was restricted to flunarizine with a treatment duration of at least four weeks; this is consistent with the recommendations of clinical guidelines for migraine prophylaxis [10–12, 39, 40]. This allows for more targeted evaluations and reduces variables regarding the different types and doses of conventional migraine prophylaxis treatment. This review also conducted subgroup analyses based on the treatment duration, follow-up duration, and dosage of flunarizine.

The major limitations of this systematic review include the low methodological quality of included studies and high heterogeneity across most meta-analyses and subgroup analyses, reducing the certainty of evidence. Future studies need to adopt more rigorous designs to ensure appropriate sequence generation, allocation concealment, and blinding procedures. It was noted that the CHM formulas were administered with different forms including decoction, pills, and granule. However, this systematic review failed to conduct subgroup meta-analysis based on CHM forms due to the small number of studies which applied same CHM formulation and reported same outcome measures. Differences of effectiveness among diverse CHM forms with the same formulation could be explored in future studies. Safety evaluation of treatments should also be given more attention in future clinical studies so that clinicians and patients will be able to make more informed decisions.

## 5. Conclusion

Cautiously, the oral CHM has the potential to act as an alternative prophylactic treatment of migraine. The results from this review show that the effects of oral CHM are, at least, equivalent to flunarizine in preventing migraine attacks in adults at EoT and EoFU, well-tolerated by participants, regardless of the treatment duration, follow-up duration, and dosage of flunarizine. However, these results need to be interpreted with caution due to the low certainty of evidence. Future studies with more rigorous designs are needed to provide more concrete evidence for stronger conclusions. This review also provides evidence for two herb pairs, Chuan Xiong plus Bai Zhi and Chuan Xiong plus Tian Ma for migraine prophylaxis. In addition, this review draws attention to the potential and need to evaluate oral CHM as an adjunct treatment to flunarizine in the prophylactic treatment of migraines.

## Figures and Tables

**Table 1:** Characteristics of included studies.

Author (year)	No. of participants randomized (I: C)	Duration of migraine (years)	Age of participants (years)	Gender (male/female)	Treatment duration, follow-up duration (days)	Dosage (mg) and frequency of flunarizine	Formula names (form)	Ingredients of formulas
Cai (2018) [69]	49: 49	NS	*I*: 36.95 ± 7.140 *C*: 37.08 ± 6.82	38/60	30, NF	10, qn	Dang Gui Si Ni Tang (decoction)	Bai Shao, Gui Zhi, Dang Gui, Tong Cao, Gan Cao, Xi Xin, and Da Zao

Cai et al. (2017) [61]	32: 28	*I*: 6.50 ± 5.36 *C*: 6.30 ± 5.24	*I*: 33.6 ± 7.62 *C*: 34.20 ± 7.21	24/36	28, NF	10, qn	Li Xu Qu Feng Tongluo Fang (decoction)	Huang Qi, Ge Gen, Dan Shen, Chuan Xiong, Bai Zhi, Man Jing Zi, Xi Xin, Tu Bie Chong, and Jiang Chan

Chen (2010) [52]	40: 30	*I*: 0.5-30 *C*: 0.83-28	*I*: 20-65 *C*: 19-62	21/47	28, NF	5, qn	Shao Zhi Zhen Tong Fang (decoction)	Chuan Xiong, Dang Gui, Jiang Chan, Quan Xie, Di Long, Bai Zhi, and Gan Cao

Dai (2005) [53]	36: 34	*I*: 5.70 *C*: 5.40	*I*: 35.6 *C*: 37.2	22/48	90, 60	10, qn	Xue Sai Tong soft capsule	San Qi

Du (2014) [73]	44: 44	*I*: 6.18 ± 5.06 *C*: 6.30 ± 5.24	*I*: 44.93 ± 13.49 *C*: 48.27 ± 11.22	22/64	90, NF	5, qn	Xiong Zhi Jian Fang (decoction)	Chuan Xiong, Bai Zhi, Bai Shao, Ge Gen, Xi Xin, Man Jing Zi, Xia Ku Cao, Bo He, Gao Ben, Chai Hu, Ju Hua, and Fang Feng

Du et al. (2011) [47]	50: 50	*I*: 3.80 ± 2.30 *C*: 3.90 ± 2.40	*I*: 40.5 ± 10.6 *C*: 39.4 ± 10.3	22/78	28, NF	10, qn	Tong Xin Luo capsule	Ren Shen, Shui Zhi, Quan Xie, Tu Bie, Chong, Wu Gong, Chan Lian, Suan Zao Ren, and Bing Pian

Fu (2015) [64]	40: 40	*I*: 3.40 ± 2.10 *C*: 4.10 ± 2.30	*I*: 43.4 ± 2.7 *C*: 42.6 ± 2.5	42/38	90, NF	5, qn	Qu Feng Huo Xue Fang (decoction)	Chuan Xiong, Niu Xi, Bai Ji Li, Gan Cao, Gao Ben, Tian Ma, Bai Zhi, Gou Teng, Ge Gen, and Chai Hu

Gao et al. (2006) [57]	40: 42	*I*: 3.20 ± 1.30 *C*: 3.00 ± 1.40	*I*: 35.4 ± 4.6 *C*: 36.1 ± 6.8	32/50	60, 365	5, qd	Tian Shu capsule	Chuan Xiong and Tian Ma

Gao et al. (2009) [58]	29: 27	*I*: 9.75 ± 5.53 *C*: 8.96 ± 6.56	*I*: 41.4 ± 19.56 *C*: 42.5 ± 18.42	0/56	30, 60	5∼10, qn	NS (decoction)	Sha Ren, Chi Shao, Bai Shao, Shi Jue Ming, Mai Dong, Ju Hua, Tao Ren, Ji Xue Teng, Ye Jiao Teng, Quan Xie, and Wu Gong

Gou and Miao (2014) [74]	30: 30	NS	26∼65	37/23	90, NF	10, qn	San Pian Tang (decoction)	Chuan Xiong, Bai Zhi, Bai Shao, Bai Jie Zi, Xiang Fu, Chai Hu, Yu Li Ren, and Gan Cao

Huang et al. (2006) [65]	30: 30	*I*: 10.50 ± 4.60 *C*: 9.80 ± 3.70	*I*: 35.3 ± 3.6 *C*: 37.1 ± 3.5	22/38	30, NF	5, qn	NS (decoction)	Quan Xie, Di Long, Tian Ma, Chuan Xiong, Wu Gong, and Jiang Chan

Liang (2015) [46]	120: 120	*I*: 6.01 ± 3.75 *C*: 6.16 ± 3.20	*I*: 35.35 ± 10.87 *C*: 34.01 ± 9.06	53/170	56, 28	10, qn	He Jie Zhi Tong Fang (decoction)	Chai Hu, Chuan Xiong, Huang Qin, Ban Xia, Dang Shen, Bai Zhu, Gan Cao, Long Gu, yuan zhi, Quan Xie, and Wu Gong

Liu (2009) [48]	40: 40	NS	*I*: 44.47 ± 11.21 *C*: 42.77 ± 9.53	22/51	30, NF	10, qn	Tou Tong Fang (decoction)	Huang Lian, zhi Ban Xia. Chen Pi, Zhi Shi, Dan Nan Xing, Zhu Ru, Shi Chang Pu, Mo Han Lian, Quan Xie, Man Jing Zi, Bai Zhi, and Chuan Xiong

Luo and Shu (2013) [79]	32: 32	*I*: 4.50 *C*: 4.80	*I*: 25–60 *C*: 24–59	25/39	28, NF	5, bid	Tzu Tong Tang (decoction)	Tian Ma, Gou Teng, Shi Jue Ming, Ju Hua, Chuan Xiong, Bai Zhi, Man Jing Zi, Quan Xie, and Di Long

Ma (2014) [59]	30: 30	NS	*I*: 42.90 ± 11.74 *C*: 46.97 ± 12.29	31/29	28, NF	10, qn	Chai Hu Gui Zhi Gan Jiang Tang (decoction)	Chai Hu, Gui Zhi, Gan Jiang, Ban Xia, Huang Qin, Dang Shen, Fu Ling, and Gan Cao

Mao et al. (2011) [49]	20: 20	*I*: 11.05 *C*: 9.19	*I*: 39 *C*: 42.6	6/32	30, NF	5, qn	Chai Shao Zhi Tong Fang (decoction)	Chai Hu, Bai Shao, Bai Zhu, Dang Gui, Chuan Xiong, Qing Feng Teng, Zhi Ke, and Gan Cao

Niu et al. (2003) [56]	35: 35	NS	NS	NS	30, NF	5, qn	Yang Xue Qing Nao Ke Li (granule)	Dang Gui, Chuan Xiong, Shu Di, Zhen Zhu Mu, Jue Ming Zi, Xia Ku Cao, and Bai Shao

Pan et al. (2015) [54]	30: 30	*I*: 7.10 *C*: 6.50	*I*: 47.52 *C*: 51.38	27/33	90, 90	5∼10, qn	Xiong Long Tang (decoction)	Yan Hu Suo, Chuan Xiong, Ge Gen, Bai Zhi, Tao Ren, Di Long, Niu Xi, Hong Hua, and Xi Xin

Peng (2017) [75]	38: 38	*I*: 5.40 ± 1.80 *C*: 5.00 ± 1.50	*I*: 36.2 ± 4.2 *C*: 35.5 ± 4.1	48/28	30, NF	10, qn	1. Tong Qiao Huo Xue Tang (decoction) 2. Tian Ma Gou Teng Yin (decoction) 3. Qi Ju Di Huang Tang (decoction) 4. Ren Shen Yang Rong Tang (decoction)	1. San Qi, Chuan Xiong, Fu Shen, Chi Shao, Dan Shen, Tao Ren, Bai Zhi, Yu Jin, and Chen Pi 2. Yi Mu Cao, Tian Ma, Zhi Zi, Niu Xi, Di Long, Gou Teng, Huang Qin, Shi Jue Ming, Sang Ji Sheng, and Du Zhong 3. Gou Qi, Mo Han Lian, Nv Zhen Zi, Ju Hua, Shan Zhu Yu, Shan Yao, Shu Di, Fu Ling, Ze Xie, and Mu Dan Pi 4. Chen Pi, Huang Qi, Bai Shao, Ren Shen, Shu Di, Bai Zhu, Chuan Xiong, Dang Gui, and Gan Cao

Qian and Yan, (2006) [60]	57: 60	*I*: 5.30 *C*: 5.10	*I*: 42.5 ± 6.12 *C*: 41.5 ± 7.36	47/70	28, NF	10, qn	Tong Qiao Huo Xue Tang (decoction)	Chi Shao, Chuan Xiong, Tao Ren, Hong Hua, Bai Zhi, Cong, Sheng Jiang, Da Zao, Huang Jiu, Dan Shen, and Huang Qi

Qu et al. (2010) [67]	31: 32	NS	*I*: 41.52 ± 8.6 *C*: 42.31 ± 7.3	20/43	30, NF	5, qn	Yi Li Tian Kan Tang (decoction)	Ba Ji Tian, Zhen Zhu Mu, Rou Gui, Huang Lian, Gui Zhi, Bai Shao, Fu Ling, Chai Hu, Zhi Zi, and Wu Mei

Shen et al. (2016) [50]	60: 60	*I*: 4.62 ± 2.48 *C*: 5.05 ± 3.05	*I*: 26.98 ± 4.6 *C*: 25.54 ± 4.35	40/75	28, 28	5, qn	San Pian Tang (decoction)	Chuan Xiong, Bai Shao, Chai Hu, Yu Li Ren, Xiang Fu, Bai Zhi, Gan Cao, Bai Jie Zi, Chi Shao, and Jiang Chan

Song (2017) [68]	32: 32	*I*: 0.01 ± 0.01 *C*: 0.01 ± 0.01	*I*: 38.9 ± 2.5 *C*: 39.1 ± 2.4	9/55	60, NF	10, bid	1. Ban Xia Bai Zhu Tang (decoction) 2. Tong Qiao Huo Xue Tang (decoction) 3. Da Bu Yuan Jian (decoction) 4. Tian Ma Gou Teng Yin (decoction)	1. Gan Cao, Ju Hong, Man Jing Zi, Chuan Xiong, Fa Ban Xia, Tian Ma, Fu Ling, Ci Ji Li, and Bai Zhu 2. Tao Ren, Chuan Xiong, Hong Hua, Bai Zhi, Chi Shao, Yan Hu Suo, Chai Hu, Shi Chang Pu, Yu Jin, and Dan Shen 3. Dang Gui, Gou Qi Zi, Du Zhong, Shan Zhu Yu, Shan Yao, Shu Di, Dang Shen, and He Shou Wu 4. Chuan Xiong, Huang Qin, Tian Ma, Chong Wei Zi, Sang Ji Sheng, Niu Xi, Gou Teng, Ye Jiao Teng, Zhi Zi, and Shi Jue Ming

Sun and Xu (2016) [76]	60: 60	*I*: 0.17 ± 0.02 *C*: 2.03 ± 0.20	*I*: 56.26 ± 5.1 *C*: 55.60 ± 4.6	65/55	56, NF	10, qd	Chuan Xiong Cha Tiao San (decoction)	Chuan Xiong, Jin Jie, Fang Feng, Xi Xin, Qiang Huo, Bai Zhi, Bo He, and Gan Cao

Wang et al. (2012) [77]	60: 60	*I*: 5.40 ± 2.60 *C*: 6.30 ± 2.20	*I*: 38.5 ± 12.9 *C*: 39.2 ± 13.4	36/84	60, NF	10, qn	San Han Huo Yu Zhi Tong Fang (decoction)	Chuan Xiong, Sha Shen, Tu Fu Ling, Bahi Zhi, Bai Jie Zi, Quan Xie, Jin Jie, Man Jing Zi, Gan Cao, and Xi Xin

Wang (2016) [62]	60: 60	NS	*I*: 36.30 ± 9.66 *C*: 34.33 ± 10.5	31/89	56, NF	10, qn	Rou Gan Xi Feng Tang (decoction)	Bai Shao, Gou Qi Zi, He Shou Wu, Xiang Fu, Chai Hu, Tian Ma, Chuan Xiong, Huang Qin, and Gan Cao

Wang (2013) [51]	58: 52	*I*: 7.64 ± 2.34 *C*: 6.18 ± 2.7	*I*: 39.30 ± 12.97 *C*: 41.37 ± 12.03	60/50	30, NF	5, qn	NS (decoction)	Chuan Xiong, Bai Shao, He Shou Wu, Shi Jue Ming, Man Jing Zi, Dang Gui, Tian Ma, Tao Ren, Bai Zhi, and Quan Xie

Xie (2015) [55]	24: 24	NS	*I*: 35.05 ± 8.54 *C*: 33.55 ± 8.79	15/33	84, 28	5, qn	Zheng Tian Jiao Nang (capsule)	Bai Shao, Bai Zhi, Chuan Xiong, Dang Gui, Di Huang, Du Huo, Fang Feng, Fu Zi, Gou Teng, Hong Hua, Ji Xue Teng, Ma Huang, and Qiang Huo

Xin (2016) [56]	30: 30	NS	*I*: 18–65 *C*: 18–65	31/29	28, 28	10, qn	Tou Tong Fang (decoction)	Huang Qi, Ren Shen, Dang Gui, Bai Shao, Chuan Xiong, Yan Hu Suo, Xi Xin, Bai Zhi, and Shui Niu Jiao

Yuan (2017) [63]	30: 30	*I*: 3.90 ± 4.63 *C*: 4.30 ± 4.24	*I*: 42.53 ± 12.19 *C*: 41.87 ± 12.33	16/44	28, NF	10, qn	Xiao Chai Hu Tang plus Qing Kong Gao (decoction)	Chai Hu, Huang Qin, Huang Lian, Ban Xia, Ge Gen, Dan Nan Xing, Chuan Xiong, Hou Po, Chen Pi, Qiang Huo, Fang Feng, and Dang Shen

Zhang (2013) [71]	30: 30	NS	*I*: 39.30 ± 12.97 *C*: 41.37 ± 12.03	27/33	56, NF	10, qn	Zheng Tian Wan (pill)	Chuan Xiong, Dang Gui, Bai Shao, Di Huang, Gou Teng, Tao Ren, Hong Hua, Fu Zi, Du Huo, Fang Feng, Ma Huang, Ji Xue Teng, and Bai Zhi

Zhang and Sun (2019) [78]	32: 32	*I*: 8.57 ± 3.65 *C*: 6.29 ± 4.68	*I*: 49.86 ± 11.37 *C*: 50.23 ± 9.16	17/47	28, F	10, qn	Yang Xue Ping Gan Tang (decoction)	Xuan Fu Hua, Zhe Shi, Shi Gao, Dang Gui, Chuan Xiong, sheng di, Bai Shao, Shou Wu Teng, Xiang Fu, and Gan Cao

Zhang (2014) [70]	42: 42	NS	*I*: 40.4 ± 9.5 *C*: 41.2 ± 7.9	34/50	28, NF	5, qn	Tian Ma Gou Teng Yin (decoction)	Tian Ma, Chuan Xiong, Dang Gui, Zhi Zi, Niu Xi, Bai Shao, Sang Ji Sheng, Gou Teng, Shi Jue Ming, Ye Jiao Teng, Zhen Zhu Mu, and Tao Ren

Zhong et al. (2009) [45]	16: 16	*I*: 2.10 ± 0.60 *C*: 2.20 ± 0.50	*I*: 34.7 ± 6.2 *C*: 33.8 ± 7.1	11/21	20, NF	5, qn	Ping Gan Qian Yang Fang (decoction)	Tian Ma, Gou Teng, Shi Jue Ming, Mu Li, and Chuan Xiong

Zhu (2006) [72]	42: 42	NS	33.4 ± 8.5	32/52	30, NF	10, qn	Tian Ma Su capsule	Tian Ma

Note: bid, bis in die; *C*, control group; *I*, intervention group; mg, milligram; NF, no follow-up; No., number; NS, not stated; qd, quaque die; qn, quaque nocte; SD, standard deviation.

**Table 2:** Most frequently used herbs in the included studies.

Most commonly used herbs	Number. of studies	Scientific names
Chuan Xiong	31	(1) *Ligusticum chuangxiong* Hort.
Bai Zhi	18	(1) *Angelica dahurica* (Fisch. ex Hoffm.) Benth. et Hook. f. (2) *Angelica dahurica* (Fisch. ex Hoffm.) Benth. et Hook. f. var. formosana (Boiss) Shan et Yuan
Bai Shao	16	(1) *Paeonia lactiflora* Pall. (2) *Paeonia veitchii* Lynch
Gan Cao	13	(1) *Glycyrrhiza uralensis* Fisch. (2) *Glycyrrhiza inflata* Bat. (3) *Glycyrrhiza glabra* L.
Tian Ma	13	(1) *Gastrodia elata* Bl.
Dang Gui	12	(1) *Angelica sinensis* (Oliv.) Diels
Chai Hu	11	(1) *Bupleurum chinense* DC. (2) *Bupleurum scorzonerifolium* Willd.
Xi Xin	9	(1) *Asarum heterotropoides* Fr. Schmidt var. mandshuricum (Maxim) Kitag. (2) *Asarum sieboldii* Miq. var. seoulense Nakai (3) *Asarum sieboldii* Miq.
Tao Ren	9	(1) *Prunus persica* (L.) Batsch (2) *Prunus davidiana* (Carr.) Franch.
Quan Xie	8	(1) *Buthus martensii* Karsch
Gou Teng	8	(1) *Uncaria rhynchophylla* (Miq.) Miq. ex Havil.

**Table 3:** Treatment effects of all outcome measures.

Outcome	Overall analysis or subgroup analysis	Number of studies (*n*=)	Number of participants (*I*/*C*)	Estimated effects (RR or MD with 95% CI)	*I* ^2^ (%)
Frequency at EoT	Overall analysis	21	1567 (787/780)	MD: −1.23 (−1.69, −0.76)	97
	Subgroup analysis (treatment duration >60 days)	5	318 (160/158)	MD: −0.87 (−1.15, −1.15)	75
	Subgroup analysis (treatment duration = 56 or 60 days)	2	202 (100/102)	MD: −1.92 (−4.43, 0.60)	100
	Subgroup analysis (treatment duration = 28 or 30 days)	14	1047 (527/520)	MD: −1.16 (−1.55, −0.76)	88
	Subgroup analysis (flunarizine dosage at 5 mg daily)	8	574 (286/288)	MD: −1.64 (−2.65, −0.64)	99
	Subgroup analysis (flunarizine dosage at 10 mg daily)	11	877 (442/435)	MD: −0.99 (−1.25, −0.74)	75
	Subgroup analysis (studies used Chuan Xiong plus Bai Zhi)	10	793 (399/394)	MD: −1.00 (−1.41, −0.60)	90
	Subgroup analysis (studies used Chuan Xiong with Tian Ma)	4	278 (138/140)	MD: −1.34 (−3.00, 0.32)	99
Frequency at EoFU	Overall analysis	5	345 (170/175)	MD: −0.96 (−1.70, −0.21)	96
	Subgroup analysis (treatment duration > 60 days)	3	178 (90/88)	MD: −0.43 (−0.98, 0.12)	81
	Subgroup analysis (treatment duration = 28 days)	2	175 (88/87)	MD: −1.84 (−2.62, −1.05)	78
	Subgroup analysis (follow-up period = 56 or 60 days)	2	130 (66/64)	MD: −0.45 (−1.20, 0.30)	89
	Subgroup analysis (follow-up period = 28 days)	3	223 (112/111)	MD: −1.33 (−2.45, −0.20)	92
	Subgroup analysis (flunarizine dosage at 5 mg daily)	2	163 (82/81)	MD: −1.29 (−3.09, 0.52)	96
	Subgroup analysis (flunarizine dosage at 10 mg daily)	2	130 (66/64)	MD: −0.98 (−1.50, −0.46)	61
	Subgroup analysis (studies used Chuan Xiong with Bai Zhi)	4	253 (142/141)	MD: −0.99 (−2.17, 0.19)	96
Migraine days at EoT	Overall analysis	4	446 (225/221)	MD: −1.65 (−3.85, 0.54)	96
Migraine days at EoFU	Overall analysis	3	386 (195/191)	MD: −2.18 (−5.08, 0.72)	97
Pain VAS/NRS at EoT	Overall analysis	14	1038 (526/512)	MD: −1.04 (−1.67, −0.40)	96
	Subgroup analysis (San Pian Tang)	2	175 (87/88)	MD: −1.88, (−3.14, −0.62)	92
	Subgroup analysis (Zheng Tian pill/granule)	2	108 (54/54)	MD: −0.64, (−1.08, −0.20)	0
Pain VAS/NRS at EoFU	Overall analysis	2	163 (82/81)	MD: −1.56 (−3.73, 0.61)	96
Attack duration at EoT	Overall analysis	20	1495 (752/743)	MD: −2.24 (−3.18, −1.30)	92
Attack duration at EoFU	Overall analysis	3	250 (126/124)	MD: −3.60 (−8.85, 1.66)	97
Responder rate at EoT	Overall analysis	5	467 (235/232)	RR: 1.37 (1.23, 1.52)	0
Acute medication at EoT	Overall analysis	5	506 (255/251)	MD: −0.58 (−1.03, −0.13)	94
Acute medication usage at EoFU	Overall analysis	4	446 (225/221)	MD: −0.69 (−1.22, −0.15)	96
HIT-6 at EoT	Overall analysis	1	120 (60/60)	MD: −3.29 (−5.51, −1.07)	—

Note: *C*, control group; CI, confidence intervals; EoFU, end of follow-up; EoT, end of treatment; HIT-6, Headache Impact Test-6; *I*, intervention group; MD, mean difference; *n,* number; NRS, numerical rating scale; RR, risk ratio; VAS, visual analogue scale.

**Table 4:** Summary of GRADE assessment.

Outcome	Number of participants (*n*=)	Number of studies (*n*=)	Estimated effects (MD with 95% CI)	Certainty of the evidence
Migraine frequency at the end of treatment	1567	21	MD: −1.23 (−1.69, −0.76)	⊕⊕○○ Low^a,b^
Migraine frequency at the end of follow-up	345	5	MD: −0.96 (−1.70, −0.21)	⊕○○○ Very low^a,b,c^

GRADE working group grades of evidence High certainty: we are very confident that the true effect lies close to that of the estimate of the effect Moderate certainty: we are moderately confident in the effect estimate. The true effect is likely to be close to the estimate of the effect, but there is a possibility that it is substantially different Low certainty: our confidence in the effect estimate is limited. The true effect may be substantially different from the estimate of the effect Very low certainty**:** we have very little confidence in the effect estimate. The true effect is likely to be substantially different from the estimate of the effect Explanation: ^a^High risk of bias in blinding may limit the certainty of the results; ^b^high heterogeneity may limit the certainty of the results; ^c^small sample size may limit the certainty of results				

Note: CI, confidence interval; MD, mean difference; *n,* number.

**Table 5:** Summary of adverse events.

Adverse events	Number and severity reported by the CHM group	Number and severity reported by the flunarizine group
Gastrointestinal symptoms	11 mild	12 mild
Drowsiness	5 mild	7 mild
Fatigue	6 mild	11 mild
Dizziness	5 mild	2 mild
Insomnia	3 mild	3 mild
Irregular menstruation	3 mild	0
Decrease of platelet	1 mild	0
Acne	1 mild	0
Extrapyramidal symptoms	0	5 mild
Weight gain	0	9 mild
Liver dysfunction	0	1 moderate
Dry mouth	0	1 mild
All adverse events reported in treatment and follow-up phases	35 mild	50 mild and 1 moderate

Note: CHM, Chinese herbal medicine.

## Data Availability

This research is a systematic review, and all data are sourced from published articles.

## References

[1] Headache Classification Committee of the International Headache Society (2018). The international classification of headache disorders, 3rd edition. *Cephalalgia*.

[2] Steiner T. J., Stovner L. J., Birbeck G. L. (2013). Migraine: the seventh disabler. *Cephalalgia*.

[3] Saylor D., Steiner T. J. (2018). The global burden of headache. *Seminars in Neurology*.

[4] Headache Disorders-not respected, not resourced, All-Party Parliamentary Group on Primary Headache Disorders (APPGPHD), 2010

[5] Buse D. C., Greisman J. D., Baigi K., Lipton R. B. (2019). Migraine progression: a systematic review. *Headache: The Journal of Head and Face Pain*.

[6] Bigal M. E., Serrano D., Buse D., Scher A., Stewart W. F., Lipton R. B. (2008). Acute migraine medications and evolution from episodic to chronic migraine: a longitudinal population-based study. *Headache: The Journal of Head and Face Pain*.

[7] Becker W. J., Gawel M., Mackie G., South V., Christie S. N. (2007). Migraine treatment. *The Canadian Journal of Neurological Sciences*.

[8] Cooke L. J., Becker W. J. (2010). Migraine prevalence, treatment and impact: the Canadian women and migraine study. *Canadian Journal of Neurological Sciences/Journal Canadien des Sciences Neurologiques*.

[9] Ford J. H., Jackson J., Milligan G., Cotton S., Ahl J., Aurora S. K. (2017). A real-world analysis of migraine: a cross-sectional study of disease burden and treatment patterns. *Headache: The Journal of Head and Face Pain*.

[10] Scottish Intercollegiate Guidelines Network 155·Pharmacological Management of Migraine, 2018, https://www.sign.ac.uk/assets/sign155.pdf

[11] Huang T. C., Lai T. H., Treatment Guideline Subcommittee of Taiwan Headache Society Taiwan Headache Society (2017). Medical treatment guidelines for preventive treatment of migraine. *Acta Neurologica Taiwanica*.

[12] Pringsheim T., Davenport W., Mackie G. (2012). Canadian headache society guideline for migraine prophylaxis. *Canadian Journal of Neurological Sciences*.

[13] Evers S., Áfra J., Frese A. (2009). EFNS guideline on the drug treatment of migraine—revised report of an EFNS task force. *European Journal of Neurology*.

[14] Chronicle E., Mulleners W. (2004). Anticonvulsant drugs for migraine prophylaxis. *Cochrane Database of Systematic Reviews*.

[15] Stubberud A., Flaaen N. M., McCrory D. C., Pedersen S. A., Linde M. (2019). Flunarizine as prophylaxis for episodic migraine. *Pain*.

[16] Karsan N., Palethorpe D., Rattanawong W., Marin J. C., Bhola R., Goadsby P. J. (2018). Flunarizine in migraine-related headache prevention: results from 200 patients treated in the UK. *European Journal of Neurology*.

[17] de Bock G. H., Eelhart J., van Marwijk H. W. J., Tromp T. P., Springer M. P. (1997). A postmarketing study of flunarizine in migraine and vertigo. *Pharmacy World and Science*.

[18] Martínez-Lage J. M. (1988). Flunarizine (Sibelium) in the prophylaxis of migraine. An open, long-term, multicenter trial. *Cephalalgia*.

[19] Maltese A., Bucolo C. (2003). Pharmacokinetic profile of topical flunarizine in rabbit eye and plasma. *Journal of Ocular Pharmacology and Therapeutics*.

[20] Ye Q., Yan L.-Y., Xue L.-J. (2011). Flunarizine blocks voltage-gated Na^+^ and Ca^2+^ currents in cultured rat cortical neurons: a possible locus of action in the prevention of migraine. *Neuroscience Letters*.

[21] Ye Q., Wang Q., Yan L. Y. (2011). Flunarizine inhibits sensory neuron excitability by blocking voltage-gated Na^+^ and Ca^2+^ currents in trigeminal ganglion neurons. *Chinese Medical Journal*.

[22] Ayajiki K., Okamura T., Toda N. (1997). Flunarizine, an anti-migraine agent, impairs nitroxidergic nerve function in cerebral arteries. *European Journal of Pharmacology*.

[23] Wauquier A., Ashton D., Marrannes R. (1985). The effects of flunarizine in experimental models related to the pathogenesis of migraine. *Cephalalgia*.

[24] Vécsei L., Majláth Z., Szok D., Csáti A., Tajti J. (2015). Drug safety and tolerability in prophylactic migraine treatment. *Expert Opinion on Drug Safety*.

[25] Xu Q. (1996). *Herb Pairs of Chinese Medicine*.

[26] Lu W. I., Lu D. P. (2014). Impact of Chinese herbal medicine on American society and health care system: perspective and concern. *Evidence-Based Complementary and Alternative Medicine*.

[27] Boon H. S., Cherkin D. C., Erro J. (2004). Practice patterns of naturopathic physicians: results from a random survey of licensed practitioners in two US States. *BMC Complementary and Alternative Medicine*.

[28] Wardle J. L., Sibbritt D. W., Adams J. (2014). The interface with naturopathy in rural primary health care: a survey of referral practices of general practitioners in rural and regional New South Wales, Australia. *BMC Complementary and Alternative Medicine*.

[29] Meyer S. P. (2017). Naturopaths in Ontario, Canada: geographic patterns in intermediately-sized metropolitan areas and integration implications. *Journal of Complementary and Integrative Medicine*.

[30] Conde R., Corrêa V. S. C., Carmona F., Contini S. H. T., Pereira A. M. S. (2011). Chemical composition and therapeutic effects of Lippia alba (Mill.) N. E. Brown leaves hydro-alcoholic extract in patients with migraine. *Phytomedicine*.

[31] Liu Z.-K., Ng C.-F., Shiu H.-T. (2018). Neuroprotective effect of *Da Chuanxiong* formula against cognitive and motor deficits in a rat controlled cortical impact model of traumatic brain injury. *Journal of Ethnopharmacology*.

[32] Wang Y.-H., Liang S., Xu D.-S. (2011). Effect and mechanism of senkyunolide I as an anti-migraine compound from Ligusticum chuanxiong. *Journal of Pharmacy and Pharmacology*.

[33] Fu C., Yu L., Zou Y. (2012). Efficacy of *chuan xiong ding tong* herbal formula granule in the treatment and prophylactic of migraine patients: a randomized, double-blind, multicenter, placebo-controlled trial. *Evidence-Based Complementary and Alternative Medicine*.

[34] Pareek A., Suthar M., Rathore G., Bansal V. (2011). Feverfew (Tanacetum parthenium L.): a systematic review. *Pharmacognosy Reviews*.

[35] Zhou L., Chen P., Liu L. (2013). Systematic review and meta-analysis of traditional Chinese medicine in the treatment of migraines. *The American Journal of Chinese Medicine*.

[36] Shan C. S., Xu Q. Q., Shi Y. H., Wang Y., He Z. X., Zheng G. (2018). *Chuan xiong* formulae for migraine: a systematic review and meta-analysis of high-quality randomized controlled trials. *Frontiers in Pharmacology*.

[37] Xia W., Zhu M., Zhang Z. (2013). Effect of Tianshu capsule in treatment of migrane: a meta-analysis of randomized control trials. *Journal of Traditional Chinese Medicine*.

[38] Xiao Y., Yuan L., Liu Y. (2015). Traditional Chinese patent medicine for prophylactic treatment of migraine: a meta-analysis of randomized, double-blind, placebo-controlled trials. *European Journal of Neurology*.

[39] Li S. W., Li Y. S., Liu R. Z. (2011). Clinical guideline for diagnosis and management of migraine in China [article in Chinese]. *Chinese Journal of Pain Medicine*.

[40] Headache Group of Pain Science Branch of Chinese Medical Association (2016). Clinical guideline for migraine prophylaxis in China [article in Chinese]. *Chinese Journal of Pain Medicine*.

[41] Higgins J., Green S. (2011). *Cochrane Handbook for Systematic Reviews of Interventions Version 5.1.0*.

[42] Suzuki N. (2004). New international classification of headache disorders (ICHD-II). *Rinsho Shinkeigaku*.

[43] Guyatt G. H., Oxman A. D., Vist G. E. (2008). GRADE: an emerging consensus on rating quality of evidence and strength of recommendations. *BMJ*.

[44] Schünemann H., Brożek J., Guyatt G., O A., The GRADE Working Group (2013). GRADE handbook for grading quality of evidence and strength of recommendations.

[45] Zhong G., Li W., Luo Y. H. (2009). Herbs for calming liver and suppressing liver-yang in treatment of migraine with hyperactive liver-yang sydrome and its effects on lymphocyte protein expression: a randomized controlled trial. *Journal of Chinese Integrative Medicine*.

[46] Liang B. (2015). A clinical study on the treatment of migraine liver depression and spleen deficiency with detuning prescription.

[47] Du Y. X., Yang L. B., Ma J., Yang C. (2011). Effectiveness of clearing collaterals for migraine: a clinical trial report [article in Chinese]. *China Journal of Traditional Chinese Medicine and Pharmacy*.

[48] Liu T. T. (2009). Effects of “Tou Tong an Fang” for migraine with phlegm-heat: a randomized clinical trial.

[49] Mao L. J., Wu P. F., Mei L. H. (2011). Effects of the method of relieving depression and expelling Wind and clearing collaterals for migraine: a randomized controlled trial [article in Chinese]. *Chinese Journal of Integrative Medicine on Cardio/Cerebrovascular Disease*.

[50] Shen B., Yu C., Wang L. (2016). Clinical research: *San pian* decoction for migraine (blood stasis in liver meridian) [article in Chinese]. *Chinese Medicine Modern District Education China*.

[51] Wang Y. (2013). Clinical research: treating migraine with hyperactivity of liver *yang* by calming Liver *yang*, expelling Wind and clearing collaterals [article in Chinese]. *Henan Traditional Chinese Medicine*.

[52] Chen J. (2010). Clinical trial to evaluate the effectiveness of Qiong Zhi Zhen Tong prescription for migraine.

[53] Dai B. H. (2005). Effectiveness of *Xue se tong* soft capsule for migraine: a randomized controlled trial [article in Chinese]. *Chinese Journal of Integrative Medicine*.

[54] Pan P. K., Chen L., Ma D. Z., Wang X. L., Cao J. (2015). Effectiveness of modified *Xiong long* decoction for migraine: a clinical trial [article in Chinese]. *Shanxi Journal of Traditional Chinese Medicine*.

[55] Xie Y. L. (2015). Effectiveness of Zheng Tian capsule for migraine with hyperactive wind and blood stasis: a clinical trial.

[56] Xin L. N. (2016). A clinical study to evaluate the effectiveness of headache prescriptions for migraine (Qi deficiency and blood stasis).

[57] Gao H. M., Liu Y. Q., Wang S. P. (2006). Clinical trial report: effectiveness of *Tian shu* capsule for migraine [article in Chinese]. *Chinese Traditional Patent Medicine*.

[58] Gao J. Y., Sun J., Wu L. P., Meng H. (2009). Clinical research: Chinese herbal medicine for female migraine [article in Chinese]. *Journal of Emergency Traditional Chinese Medicine*.

[59] Cai M. K. (2018). Effectiveness of *Dang gui si ni* decoction for migraine: a clinical trial report [article in Chinese]. *World’s Latina Medicine Infectious Digoxin*.

[60] Cai Z. X., Chen Y., Wang C. Q., Li L., Wang Y., Hu Y. H. (2017). Clinical trial: *Li xu qu feng tong Luo* decoction for migraine without aura [article in Chinese]. *Jilin Traditional Chinese Medicine*.

[61] Du Q. (2014). Clinical research on the effects and potential mechanisms of Xiong Zhi decoction for migraine.

[62] Fu G. Y. (2015). Clinical trial: effectiveness of expelling wind and activating blood circulation method for migraine prophylaxis [article in Chinese]. *Clinical Journal of Chinese Medicine*.

[63] Gou C. G., Miao Z. G. (2014). Clinical research: *San pian* decoction for migraine prophylaxis [article in Chinese]. *Chinese Journal of Practical Nervous Diseases*.

[64] Huang H. Q., Yang R. Y., Zhao Y. X. (2006). Clinical research: the method of clearing collaterals in treating migraine [article in Chinese]. *Journal of Chinese Medicine*.

[65] Luo J. C., Shu J. S. (2013). Clinical research: *Tou tong* decoction for migraine [article in Chinese]. *International Traditional Journal of Chinese Medicine*.

[66] Ma J. D. (2014). Effectiveness of Chai Hu Gui Zhi gan Jiang decoction for migraine: a randomized controlled clinical trial.

[67] Peng Z. C. (2017). Effectiveness of Chinese herbal medicine for migraine: a clinical trial report [article in Chinese]. *El Journal of International Chinese West Medicine Cardiovascular Disease*.

[68] Qian Y. L., Yan D. (2006). Clinical trial report: effectiveness of *Tong qiao huo xue* decoction for migraine [article in Chinese]. *Hunan Journal of Traditional Chinese Medicine*.

[69] Qu M., Tang Q. S., Pei Q. H., Li X. Randomized clinical trial: the method of warming y*ang and tonifying qi* in treating migraine.

[70] Song S. Q. (2017). Effectiveness of Chinese herbal medicine for migraine based on Chinese medicine syndrome differentiation: a clinical trial report [Article in Chinese]. *Guangxi Journal of Traditional Chinese Medicine*.

[71] Sun D., Xu B. H. (2016). Clinical research on *Chuan xiong cha tiao san* for migraine and the subsequent change in the level of beta-endorphin and 5-HT [article in Chinese]. *Journal of Emergency Traditional Chinese Medical*.

[72] Wang G. F., Li W. T., Wang S. L. (2012). Clinical research on the effects of *San han huo yu zhi tong* decoction for migraine (*Yang* deficiency and cold stasis type) [article in Chinese]. *Journal of New Chinese Medicine*.

[73] Wang L. Q. (2016). Clinical trial: Rou gan Xi Feng decoction for prophylaxis of migraine (liver yang uprising type).

[74] Yuan A. Q. (2017). Clinical research: using the method of clearing heat, drying dampness and harmonizing Shaoyang for migraine.

[75] Zhang P. (2013). Clinical research: Zheng Tian pill for migraine (blood stasis type).

[76] Zhang Q. X., Sun F. X. (2019). Clinical research on the effects of *Yang xue ping gan* decoction for migraine and the subsequent change in the level of serum ET-1, CGRP and NO [article in Chinese]. *Journal of Sichuan Traditional Chinese Medicine*.

[77] Zhang Y. D. (2014). Observation on the treatment effect of treating migraine with liver *yang* uprising by calming liver *yang,* removing blood stasis and clearing collaterals [article in Chinese]. *Guidence Chinese Medicine*.

[78] Zhu Y. L. (2006). Effectiveness of *Tian ma su* capsule for migraine: a randomized controlled trial [article in Chinese]. *Publications Medicine for Magnesium*.

[79] Niu Z. P., Hou Y. L., Ren X. (2003). Effectiveness of *Yang xue qing nao* granule for migraine: a randomized controlled trial [article in Chinese]. *Integrated Chinese West Medicine Journal of Cardio-Cerebrovasc Disorder*.

[80] Chinese Pharmacopoeia Commission (2015). *Chinese Pharmacopoeia*.

[81] Schmidt R., Oestreich W. (1991). Flunarizine in migraine prophylaxis: the clinical experience. *Journal of Cardiovascular Pharmacology and Therapeutics*.

[82] Shimell C. J., Fritz V. U., Levien S. L. (1990). A comparative trial of flunarizine and propranolol in the prevention of migraine. *South African Medical Journal*.

[83] Sarchielli P., Mancini M. L., Calabresi P. (2006). Practical considerations for the treatment of elderly patients with migraine. *Drugs & Aging*.

[84] Brücke T., Wöber C., Podreka I. (1995). D2 receptor blockade by flunarizine and cinnarizine explains extrapyramidal side effects. A SPECT study. *Journal of Cerebral Blood Flow & Metabolism*.

[85] Lin W., Lin C. L., Hsu C. Y., Wei C. Y. (2019). Flunarizine induced Parkinsonism in migraine group: a nationwide population-based study. *Frontiers in Pharmacology*.

[86] Bassi P., Brunati L., Rapuzzi B., Alberti E., Mangoni A. (1992). Low dose flunarizine in the prophylaxis of migraine. *Headache: The Journal of Head and Face Pain*.

[87] Heykants J., De Crée J., Hörig C. (1979). Steady-state plasma levels of flunarizine in chronically treated patients. *Arzneimittel-Forschung*.

[88] May A., Schulte L. H. (2016). Chronic migraine: risk factors, mechanisms and treatment. *Nature Reviews Neurology*.

[89] Kang O.-H., Lee G.-H., Choi H. J. (2007). Ethyl acetate extract from Angelica Dahuricae radix inhibits lipopolysaccharide-induced production of nitric oxide, prostaglandin E2 and tumor necrosis factor-*α* via mitogen-activated protein kinases and nuclear factor-*κ*B in macrophages. *Pharmacological Research*.

[90] Sun J., Li H., Sun J., Liu H., Chen J., Wang C. (2017). Chemical composition and antimigraine activity of essential oil of Angelicae dahuricae radix. *Journal of Medicinal Food*.

[91] Wang P.-H., Zhao L.-X., Wan J.-Y. (2016). Pharmacological characterization of a novel gastrodin derivative as a potential anti-migraine agent. *Fitoterapia*.

[92] Liu Y., Gao J., Peng M. (2018). A review on central nervous system effects of Gastrodin. *Frontiers in Pharmacology*.

[93] Wang S., Hu Y., Tan W. (2012). Compatibility art of traditional Chinese medicine: from the perspective of herb pairs. *Journal of Ethnopharmacology*.

[94] Hou M., Tang Q., Xue Q. (2017). Pharmacodynamic action and mechanism of *Du Liang* soft capsule, a traditional Chinese medicine capsule, on treating nitroglycerin-induced migraine. *Journal of Ethnopharmacology*.

[95] Feng S., He X., Zhong P., Zhao J., Huang C., Hu Z. (2018). A metabolism-based synergy for total coumarin extract of radix angelicae dahuricae and Ligustrazine on migraine-modle rats. *Molecules*.

[96] Worthington I., Pringsheim T., Gawel M. J. (2013). Canadian headache society guideline: acute drug therapy for migraine headache. *The Canadian Journal of Neurological Sciences*.

[97] Tfelt-Hansen P., Pascual J., Ramadan N. (2012). Guidelines for controlled trials of drugs in migraine: third edition. A guide for investigators. *Cephalalgia*.

